# The Impact of Early Life Exposure to Cannabis: The Role of the Endocannabinoid System

**DOI:** 10.3390/ijms22168576

**Published:** 2021-08-09

**Authors:** Annia A. Martínez-Peña, Genevieve A. Perono, Sarah Alexis Gritis, Reeti Sharma, Shamini Selvakumar, O’Llenecia S. Walker, Harmeet Gurm, Alison C. Holloway, Sandeep Raha

**Affiliations:** 1 Graduate Program in Medical Sciences, McMaster University, Hamilton, ON L8S 4K1, Canada; martia77@mcmaster.ca (A.A.M.-P.); peronog@mcmaster.ca (G.A.P.); hollow@mcmaster.ca (A.C.H.); 2 Department of Obstetrics and Gynecology, McMaster University, Hamilton, ON L8S 4K1, Canada; sag8@st-andrews.ac.uk (S.A.G.); sauveo@mcmaster.ca (O.S.W.); gurmh@mcmaster.ca (H.G.); 3 Department of Pediatrics, McMaster University, Hamilton, ON L8S 4K1, Canada; reeti.sharma@mail.utoronto.ca (R.S.); shamini.selvakumar@medportal.ca (S.S.)

**Keywords:** cannabis, Δ^9^-THC, pregnancy, placenta, endocannabinoid system, fetal development, reproductive health

## Abstract

Cannabis use during pregnancy has continued to rise, particularly in developed countries, as a result of the trend towards legalization and lack of consistent, evidence-based knowledge on the matter. While there is conflicting data regarding whether cannabis use during pregnancy leads to adverse outcomes such as stillbirth, preterm birth, low birthweight, or increased admission to neonatal intensive care units, investigations into long-term effects on the offspring’s health are limited. Historically, studies have focused on the neurobehavioral effects of prenatal cannabis exposure on the offspring. The effects of cannabis on other physiological aspects of the developing fetus have received less attention. Importantly, our knowledge about cannabinoid signaling in the placenta is also limited. The endocannabinoid system (ECS) is present at early stages of development and represents a potential target for exogenous cannabinoids in utero. The ECS is expressed in a broad range of tissues and influences a spectrum of cellular functions. The aim of this review is to explore the current evidence surrounding the effects of prenatal exposure to cannabinoids and the role of the ECS in the placenta and the developing fetus.

## 1. Introduction

With a long-recorded history of human use, cannabis is one of the most widely used psychoactive drugs worldwide. In the past decade, cannabis use has grown rapidly, particularly in developed countries [1]. In fact, in 2018 Canada became the first developed nation to legalize cannabis for recreational use. According to the National Cannabis Survey, approximately 25% of Canadians of reproductive age (15 to 44) reported using cannabis products in the prior three-month period. Among the users, more than half reported using some form of cannabis daily (40%) or weekly (17%) [2].

The legalization of cannabis may impact public perceptions regarding the risks and benefits of cannabis and its constituents. Of particular concern is cannabis use during pregnancy, where it is considered to be the most commonly used illicit drug [3,4,5,6,7]. Ko and co-workers reported that 70% of pregnant and non-pregnant women in the US believe that there is little or no harm in using cannabis once or twice per week [8]. It is more commonly used during the first trimester for its antiemetic properties in mitigating nausea associated with morning sickness [9,10,11,12]. However, in some cases it is also used to relieve pain and to aid with disorders such as anxiety and depression throughout pregnancy [13]. Moreover, a longitudinal prospective study in the UK discovered that 48% of women who used cannabis in the year prior to their pregnancy continued to smoke throughout gestation [14].

Few Canadian studies have investigated the prevalence of marijuana use during pregnancy. A retrospective cohort study assembled from the Better Outcomes Registry & Network (BORN) (Ontario, Canada) database reported a 61% increase in the overall prevalence of cannabis use during pregnancy between 2012 and 2017. In fact, prevalence was highest among women aged 15–24 years old, who reported an increase in usage from 4.9% in 2012 to 6.5% in 2017 [15]. Similarly, in the US, the 2019 National Survey on Drug Use and Health revealed that approximately 5.4% of pregnant women reported using cannabis during the past month [16]. Data from the Screening for Pregnancy Endpoints (SCOPE) study, which includes nulliparous women with singleton pregnancies between 2004 and 2011 from Australia, New Zealand, Ireland, and the UK, revealed that self-reported cannabis use during pregnancy was approximately 4% [17]. These studies reveal a general increase in the use of cannabis during pregnancy in developed countries. Additionally, given that most studies rely solely on self-reports, the actual prevalence of cannabis use may be underestimated among pregnant women [18,19]. It is interesting to note that these numbers persist despite the advice of the Society of Obstetricians and Gynecologists of Canada [20], and the fact that most Canadian dispensaries (93%) recommend against the use of cannabis during pregnancy, according to a recent study [21]. Furthermore, while there is evidence that supports the physiological benefits of cannabis-derived products, particularly in the treatment of chronic pain, spasticity, sleep disorders, nausea and vomiting [22], there is very little research that addresses these benefits during pregnancy or the potential effects of non-psychoactive components when consumed on their own [23]. In considering the consequences of cannabis exposure, it is important to address the changes in cannabis composition over the last decades. The cannabis plant contains more than 500 compounds from several chemical classes including cannabinoids (phytocannabinoids), mono- and sesquiterpenes, sugars, hydrocarbons, flavonoids, steroids, nitrogenous compounds, amino acids, and simple fatty acids [24,25]. Among these, the phytocannabinoid delta-9-tetrahydrocannabinol (Δ^9^-THC) is one of the most studied constituents, as it is the major cannabinoid present in most cannabis products and is known for its psychoactive properties [26,27,28]. In fact, studies that have analyzed the concentrations of Δ^9^-THC over time describe increases in the proportion of this compound in cannabis in recent decades [29,30,31,32,33]. According to Health Canada, Δ^9^-THC potency in dried cannabis has increased from an average of 3% in the 1980s to around 15% in 2019, with some strains possessing as high as 30% Δ^9^-THC [34]. This considerable increase in cannabis potency may result in different effects on human health from those observed in studies carried out several decades ago.

Efforts to understand the mechanism of action of Δ^9^-THC led to the identification of the endocannabinoid system (ECS), which consists of cannabinoid receptors, endocannabinoids and their metabolic enzymes [35]. In addition to participating in the modulation of the neurological, immunological and endocrine systems [36], there is accumulating evidence that highlights the role of the ECS in reproductive processes such as fertilization, implantation, embryonic development and placental growth [37,38,39,40]. Therefore, exposure to insults that can disturb ECS signaling may lead to negative reproductive and pregnancy outcomes.

**Figure 1:**
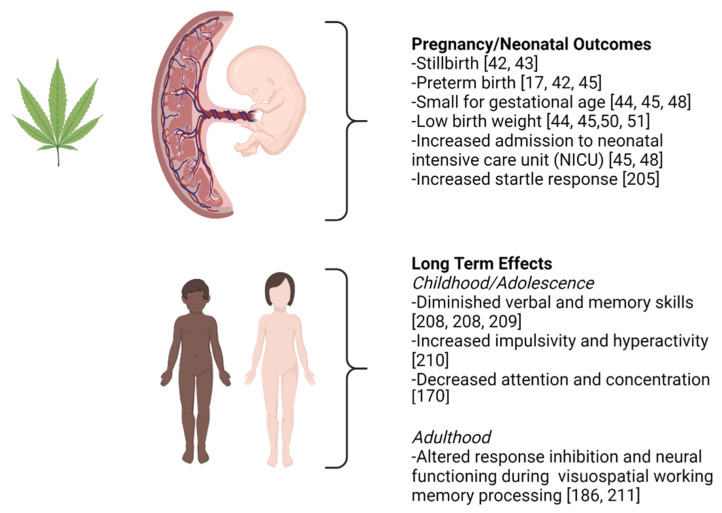
**The effects of cannabis on human pregnancy outcomes as well as longer term effects postnatally are summarized**. In considering the outcomes summarized here, it should be noted that the cannabis dosing in many of the studies in human participants is self-reported. Greater details about these studies can be found in body of this review. This figure was created using BioRender.com accessed on 5 August 2021.

Overall, reports about the effects of cannabis use during pregnancy in humans are conflicting [41]. While many researchers have demonstrated that prenatal cannabis use is associated with stillbirth [42,43], preterm birth [17,42,44,45,46], small for gestational age [44,45,47,48], low birth weight [44,45,49,50,51,52], and increased admission to neonatal intensive care units [45,48,52] (see Figure 1), others have reported no association between prenatal cannabis use and adverse pregnancy or neonatal outcomes [53,54,55]. Inconsistent conclusions from maternal cannabis studies in humans could be a result of confounding variables related to socio-demographics, sample size, maternal nutrition, poly-substance use, cannabis potency and frequency and duration of use; especially when much of this data relies on self-reported use. Another important variable worth considering is the method of consumption. While the most common method of cannabis consumption is smoking, other forms have gained popularity in recent years [56]. However, although potency and pharmacokinetic properties may differ between them, the active ingredients remain the same and the developing fetus may still be exposed. There is currently not enough data to support that any consumption method is safer than others.

Animal studies have shown that prenatal exposure to cannabis, or Δ^9^-THC specifically, results in increased resorptions [57], increased number of stillbirths [58], low birth weight [59,60,61], reduced fetal to placental weight ratio [59,61], decreased brain to body weight ratio [61], decreased liver to body weight ratio [61], and decreased pancreatic weight at birth [60]. In addition to the effects observed during pregnancy and immediately after birth, prenatal exposure to cannabis may also result in long-term alterations in the offspring’s health. The “Double Hit Hypothesis” is a phenomenon that has been used to describe the effects of other neurodevelopmental teratogens. It has been proposed that exposure to cannabis during early stages of development may deliver the “first hit” to the fetal endocannabinoid system but may not always result in immediate observable effects. In fact, the first hit increases susceptibility to neurodevelopmental deficits in adult offspring following exposure to postnatal environmental stressors (“second hit”), such as tobacco smoke and other illicit drugs and pollutants [62]. 

Taking these studies into account, the goal of this review is to discuss the role of the endocannabinoid system during pregnancy and the effects associated with prenatal exposure to cannabinoids in animal and human studies. Importantly, this review aims to highlight the role of the ECS during fetal development and the possible long-term consequences of its disruption. A comprehensive search was conducted on PubMed using the following key words: cannabis, cannabinoids, Δ^9^-THC, pregnancy, endocannabinoid system, fetal, placenta, metabolism, reproduction. Relevant literature was included, and references were used to find other related sources.

## 2. The Endocannabinoid System

The endocannabinoid system is a molecular signaling pathway that regulates several physiological processes including pain, inflammation, neurodevelopment, appetite, stress, metabolism and reproduction (reviewed in [63,64,65,66,67]). The ECS consists of cannabinoid receptors (CB), cannabinoid ligands (i.e., endocannabinoids), membrane transporters and the metabolic enzymes that modulate endocannabinoid synthesis and breakdown [66,68].

### 2.1. ECS Ligands

Endocannabinoids are naturally occurring lipid mediators that include amides, esters and ethers of long chain polyunsaturated fatty acids [69]. The primary endocannabinoids associated with the signaling events in the various physiological systems indicated above are anandamide (N-arachidonoylethanolamine, AEA) and 2-arachidonoyl glycerol (2-AG) [70,71]. AEA can be synthesized from *N*-arachidonoyl phosphatidyl ethanol (NAPE) through four different pathways that may involve one or more enzymes: (1) NAPE-phospholipase D (NAPE-PLD); (2) NAPE-phospholipase C and phosphatase; (3) alpha/beta domain-containing hydrolase 4 (ABHD4) and glycerophosphodiesterase; or (4) ABHD4 and lyso-NAPE-PLD [68]. Typically, 2-AG is synthesized from phosphatidyl inositol bisphosphate by phospholipase C (PLC) and diacylglycerol lipase (DAGL), although synthesis via phospholipase A and lypho-PLC has also been proposed [68,69,72]. While other endocannabinoids like virodhamine, 2-arachidonoyl glycerol ether and N-arachionoyl dompamine exist [68,73], less is known about the pharmacology and their roles in cellular signaling.

For years, it was generally accepted that endocannabinoids were synthesized on demand from membrane phospholipid precursors [69]; however, recent studies suggest that these compounds may be stored within intracellular lipid droplets (adiposomes), protracting their effects on downstream receptors [74,75].

The cannabis plant has numerous bioactive phytochemicals, including over 120 cannabinoids [68]. The best characterized phytocannabinoids are Δ^9^-THC and cannabidiol (CBD). Indeed, while the route of administration and variability within and between subjects influence the pharmacokinetics of these compounds, both Δ^9^-THC and CBD have been detected in the lungs, heart, brain, liver, adipose tissue, and breast milk, and can readily cross the placenta [76]. Δ^9^-THC is the most potent psychoactive component found in cannabis extracts that causes a state of euphoria (generically referred to as the “high”) and possesses therapeutic utility, treating nausea and emesis, appetite, spasticity, pain, and anxiety [77,78]. These effects are largely attributed to agonist activity at CB1 and CB2 receptors. Δ^9^-THC is metabolized into its active metabolite, 11-hydroxy-Δ^9^-THC, and inactive metabolite, 11-carboxy-Δ^9^-THC, which are readily excreted in the feces and urine, respectively [76]. CBD is another phytocannabinoid that modulates pain, spasticity, and inflammation, while lacking the psychoactive properties typically seen with Δ^9^-THC. In fact, CBD is thought to have a protective effect as co-administration of CBD with Δ^9^-THC has been shown to alleviate the psychotic effects of Δ^9^-THC by allosterically modulating and indirectly antagonizing CB receptors [77,79]. While CBD can be metabolized into various derivatives of 7-carboxy-CBD, most CBD is excreted in the feces unchanged [80]. Due to the medicinal efficacy of cannabis, Δ^9^-THC and CBD, many synthetic analogues have been synthesized to mimic the benefits of these cannabinoids including WIN-55,212, JWH-018, JWH-122, UR-144, CP55940, ajulemic acid, dronabinol and HU308 [77,81]. 

### 2.2. ECS Signaling

The effects of cannabinoids are mainly mediated via CB1 and CB2 activation. While both isoforms are ubiquitously expressed throughout the body, CB1 is found predominantly in the central nervous system [82], while CB2 is found in the periphery within immune cells such as B lymphocytes and macrophages [83,84,85]. Both CB1 and CB2 are G protein-coupled receptors that modulate several signaling pathways. Most cannabinoid receptors are coupled to G_i/o_ protein subunits which inhibit adenylyl cyclase activity, decrease intracellular cyclic adenosine monophosphate (cAMP) levels and protein kinase A (PKA) phosphorylation, thus perturbing downstream PKA-regulated events [81]. Additionally, some CB1 receptors are localized within intracellular structures such as endosomes, lysosomes and mitochondria. These subcellular CB1 receptors function to mediate β-arrestin signaling, internal calcium stores, permeability of lysosomes and mitochondrial respiration and cAMP production [86]. AEA and Δ^9^-THC are partial agonists with high affinity to CB1/CB2, while 2-AG is a full agonist at both receptors with moderate affinity [81]. Contrastingly, CBD has been proposed to function as an antagonist and has weak CB receptor affinity [77]. Some synthetic cannabinoids were created to be more potent than AEA, 2-AG and Δ^9^-THC, and possess greater affinity and efficacy at cannabinoid receptors (reviewed in [81]). Alternatively, AEA, 2-AG, Δ^9^-THC, CBD and synthetic cannabinoids can also mediate their effects independent of CB1/CB2 through the orphan receptor, G protein-coupled receptor 55 (GPR55) [87,88]. GPR55 couples to Gα_12/13_ and G_q_ proteins which signal through Ras homolog gene family member A (RhoA) and PLC pathways to increase intracellular Ca^2+^ [89,90]. GPR55 is expressed in several regions of the brain, liver, pancreatic β-cells, gastrointestinal tract, and adipose tissue, playing a role in regulating neural development, emotion, cognition, and energy homeostasis [90,91,92]. Additionally, AEA and (to a lesser extent) 2-AG have been shown to activate the non-selective cation channel transient receptor potential vanilloid 1 (TRPV1) [93,94,95]. TRPV1 is the cognate receptor for capsaicin, though other harmful stimuli like heat and acidic toxins can activate this receptor as it modulates pain, nociception, and temperature sensing [73]. Consequently, expression of TRPV1 is predominantly within sensory neurons where it has been found to colocalize with cannabinoid receptors [96,97]. Finally, activation of peroxisome proliferator activated receptor (PPAR) superfamily of nuclear receptors by cannabinoids modulates several physiological processes including energy homeostasis and metabolism, inflammation, neuroprotection, epilepsy, addiction, the circadian rhythm, and cognition [98].

Multiple pathways have been reported regarding termination of endocannabinoid signaling of AEA and 2-AG [66,81]. Hydrolysis of AEA and 2-AG is primarily regulated by fatty acid amino hydrolase (FAAH) and monoacylglycerol lipase (MAGL), respectively [81]. Additionally, the arachidonic acid signature of the AEA and 2-AG compounds allows for these endocannabinoids to function as congeners of arachidonic acid and thus serve as substrates for cyclooxygenase-2 (COX2), lipoxygenase (LOX) and cytochrome (CYP) 450 metabolism [81]. Consequently, there is potential for crosstalk between endocannabinoid and eicosanoid signaling pathways. Activation of COX2 leads to the formation of neutral prostaglandin derivatives, prostamides (prostaglandin-ethanolamide) and prostaglandin-glyceryl esters while LOX converts endocannabinoids into hydroxyeicosatetranoic acids (HETEs) and CYP450 converts into both HETEs and epoxyeicosatrienoic acids (EETs) [99]. Although COX2-derived endocannabinoid metabolites exert little to no activity on cannabinoid or prostanoid receptors, HETEs and EETs may bind to cannabinoid receptors and enhance or diminish endocannabinoid signaling [99,100].

## 3. The ECS and the Placenta

In females, the expression of ECS components has been identified in reproductive tissues including the ovary [66,101], follicular fluid [102], embryo [103], uterus [104,105] and placenta [106]. The ECS plays a crucial role in early human development, participating in processes such as gametogenesis, embryo implantation, neurodevelopment, peripheral organogenesis, and postnatal development [40,107]. In vitro experiments have demonstrated that exposure of early embryos to high levels of synthetic cannabinoids, phytocannabinoids and endocannabinoids inhibits blastocyst formation, zonal hatching, and trophoblastic differentiation [103,108,109,110,111].

The placenta is a transient organ, composed of a variety of cell types, that is critical for proper fetal development and pregnancy success. Trophoblasts are specialized placental cells that facilitate the attachment of the conceptus to the uterine wall and predominantly constitute the maternal–fetal interface [112]. As such, trophoblasts play an important role in supporting nutrient and gas exchange, endocrine signaling, protein biosynthesis and fetal protection during pregnancy [112,113]. The best characterized trophoblast subtypes are the syncytiotrophoblast (ST) and extravillous trophoblast (EVT) cells, both of which are derived from cytotrophoblast (CT) progenitor cells [113]. ST form a tightly arranged multinucleated layer around the chorionic villi, which is responsible for regulating transmission of substances between the mother and the developing fetus, as well as protein biosynthesis [113]. The EVTs are responsible for migrating away from the placenta and invading the endometrial stroma and the lumen of maternal spiral arteries [112]. 

The ECS plays an important role in the modulation of placental development and pregnancy (reviewed in [113,114,115,116]). First trimester trophoblasts and term placental tissues have been shown to express CB1 and CB2 receptors, suggesting that the placenta may be a target for cannabinoids [37,106,117,118]. Similarly, expression of the ECS metabolic enzymes, NAPE-PLD, FAAH, DAGL and MAGL has been shown in primary CT, EVT and ST isolated from first trimester and term placentas, as well as in BeWo cells which are an in vitro model for placental CT [37,117,119,120,121,122,123]. To date, only AEA has been measured in the human placenta [124], while 2-AG has been measured in the placenta of baboons (*Papio* spp.) [125] and rats [126]. Expression of other cannabinoid receptors, TRPV1 and GPR55, have also been described in the placenta, where TRPV1 was localized in CT and ST, and GPR55 was identified in the placental endothelium [127,128]. 

### 3.1. Altered ECS Signaling in the Placenta

**Figure 2:**
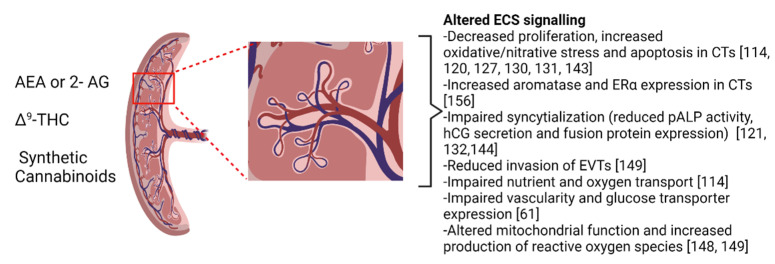
**Cannabinoid’s impact signaling in the placenta**. The reported effects of anandamide (AEA), 2-arachidonoyl glycerol (2-AG), delta-9-tetrahydrocannabinol (Δ^9^-THC) and synthetic cannabinoid agonists have demonstrated that endocannabinoid system (ECS) signaling impacts a broad range of cellular functions within the placenta including estrogen receptor alpha (ERα) expression, placental alkaline phosphatase (pALP) activity and human chorionic gonadotropin (hCG) secretion in cytotrophoblasts (CT) or extravillous trophoblasts (EVT). The magnified inset illustrates a single spiral artery assembly around which the maternal-fetal interface is constructed. Many of the changes outlined in this figure occur at the maternal fetal interface. Greater details about these studies can be found in the body of this review. This figure was created using BioRender.com accessed on 5 August 2021.

Placentation involves continuous tissue remodeling and requires proper trophoblast turnover (i.e., tightly regulated proliferation, differentiation, and apoptosis) [113]. The role of the ECS in modulating trophoblast proliferation and apoptosis, syncytialization, migration and invasion, protein biosynthesis and transport of nutrients to the fetus has been extensively reviewed [114,129]. In mice, genetic ablation of CB1 inhibited trophoblast proliferation, differentiation, and invasiveness, significantly impairing placental development [122]. This suggests that aberrant placental ECS signaling may impair pregnancy success. Previous studies have shown that AEA and 2-AG exposure significantly decreases cell viability and proliferation, and induces oxidative/nitrative stress and apoptosis via TRPV1 in primary CTs and via CB receptors in BeWo cells [120,127,130] (see Figure 2). Recently, Almada and colleagues proposed that 2-AG-induced oxidative stress and apoptosis may be mediated through CB2 activation and induction of endoplasmic reticulum stress and protein kinase RNA-like endoplasmic reticulum kinase/eukaryotic initiation factor 2/activating transcription factor 4 C/EBP homologous protein (PERK/eIF2a/ATF4/CHOP) signaling pathway [131].

Additionally, disruption in ECS signaling has been associated with changes in syncytialization and ST function. Exposure to 2-AG has been previously shown to reduce placental alkaline phosphatase (pALP) activity, human chorionic gonadotropin (hCG) secretion, and mRNA expression of fusion proteins (glial cell missing-1 and syncytin), demonstrating impaired syncytialization [121]. Exposure to AEA similarly dysregulated syncytialization and altered expression of fusion proteins and hCG secretion [132]. Both AEA and 2-AG disrupt protein biosynthesis and endocrine function in STs, as well as impair the transport of nutrients, oxygen and other substances to the fetus, effects which may be attributed to activity at CB1 and CB2 receptors (Reviewed in: [114]) (see Figure 2). A recent study in placental explants and BeWo cells demonstrated that AEA impaired ST function by altering the expression of efflux transporter proteins (breast cancer resistance protein, BCRP/ABCG2) which provide fetal protection against xenobiotic exposure [133]. These observations were reversed following treatment with CB2 antagonist (AM630) or cAMP analog (8-Br-cAMP), suggesting that AEA mediates placental transporter expression via CB2-cAMP signaling [133]. Together, these in vitro studies suggest the importance of ECS signaling in mediating trophoblast turnover and proper placentation. 

Abnormal placentation has been shown to contribute to many pregnancy complications including spontaneous/recurrent miscarriage, preeclampsia, fetal growth restriction, and still-birth (reviewed in [116,129]). However, investigation into the role of the ECS in placental-related pregnancy complications is limited. There are reports that plasma AEA levels oscillate during pregnancy, with low levels throughout gestation and increased levels during labor, suggesting that AEA may play a critical role in parturition [134]. Interestingly, FAAH has been shown to modulate local levels of AEA in the placenta [37,123] and has been linked to early pregnancy success [117]. Studies conducted in women who miscarried in their first trimester showed that FAAH expression and activity significantly decreased in placental trophoblasts, as well as maternal lymphocytes [123,135]. Similarly, reductions in circulating FAAH corresponded to increases in circulating AEA levels of patients who underwent in vitro fertilization (IVF) embryo transfer and failed to achieve pregnancy in contrast to patients who became pregnant [136]. These findings suggest that tightly regulated maternal AEA levels and FAAH activity are needed for the establishment of pregnancy. In contrast, preeclamptic patients demonstrated reduced plasma AEA levels during pregnancy, an effect that was also attributed to alterations in the enzymatic activity of FAAH and NAPE-PLD [116,137]. However, there are studies that report opposing expression profiles of these metabolic enzymes in preeclamptic patients [119,138]. Nonetheless, these findings demonstrate that aberrant ECS signaling in the placenta is detrimental to the maintenance of pregnancy and there appear to be critical windows for ECS disruption by exogenous cannabinoids which can contribute to adverse pregnancy and fetal outcomes.

### 3.2. Impact of Exogenous Cannabinoids on the Placenta and ECS Signaling 

There is compelling evidence that exposure to exogenous cannabinoids affects pregnancy outcome and fetal development [139]. In fact, Δ^9^-THC in the plasma of pregnant mothers can readily cross the placenta in both humans and animals [140,141,142] and may compromise placentation. Gestational exposure to Δ^9^-THC has been shown to result in placental insufficiency in rats, an effect attributed to impaired labyrinth-specific maternal-fetal vascularity and glucose transporter expression [61]. While it is plausible that Δ^9^-THC-induced defects in placentation can lead to adverse pregnancy outcomes such as symmetric fetal growth restriction [61], the exact mechanisms by which these obstetrical complications occur is poorly understood.

Exogenous cannabinoids have also been shown to impair placentation via aberrant trophoblast proliferation, differentiation, and apoptosis. Exposure to 10–20 μM Δ^9^-THC significantly decreased BeWo cell proliferation and viability, as well as altered the expression of genes involved in cell growth, apoptosis, cell morphology and ion exchange [143,144]. In primary CT and ST cells, high doses of Δ^9^-THC (>50 μM) also decreased cell viability independent of CB receptor-mediated signaling [145]. In line with this effect, treatment with synthetic cannabinoids WIN-55,212, JWH-018, JWH-122 and UR-144 has been shown to induce apoptotic cell death and increase caspase 3/7 and 9 activity via CB activation (except JWH-122, which is CB-independent). Additionally, WIN-55,212, UR-144 and JWH-122 caused loss of mitochondrial membrane potential, while JWH-018 and JWH-122 increased reactive oxygen species (ROS) production [146,147] (see Figure 2). Furthermore, Δ^9^-THC has been shown to increase the expression of endoplasmic reticulum stress markers and CHOP via CB1 and CB2 signaling, and lead to mitochondrial injury [148]. Similarly, impairment of mitochondrial function following Δ^9^-THC exposure has also been observed in parallel with reduced syncytialization of BeWo cells and reduced invasion of the EVT model cell line, HTR8/SVneo cells, critical processes for early establishment and maintenance of the placenta [144,149].

The transport of important nutrients, gas and substances between the mother and developing fetus is critical for pregnancy success. Disruption in placental uptake of key nutrients may result in defective placentation and fetotoxicity. Chronic exposure to Δ^9^-THC has been shown to alter trophoblast expression of transporter proteins and uptake of folic acid, which is an important micronutrient necessary for normal placental and fetal development [150,151]. CBD is another potent phytocannabinoid that has been shown to treat nausea, insomnia, anxiety, and pain while lacking the psychological and euphoric effects of Δ^9^-THC [23]. Despite the therapeutic utility for CBD to treat pregnancy-related symptoms, very little is known regarding the safety of CBD use during pregnancy or the impact of CBD on placental development and ECS signaling [23,152]. One study conducted by Feinshtein and colleagues showed that in vitro and ex vivo CBD exposure significantly increased placental barrier permeability via altered breast cancer resistance protein function, an important placental transporter that mediates efflux of xenobiotic compounds [153]. This finding suggests CBD exposure during pregnancy may increase fetal susceptibility to other damaging constituents found in cannabis-related products [153]. Moreover, the placenta is also responsible for the synthesis and secretion of steroid hormones and other endocrine factors that support pregnancy [154]. Perturbations to estrogen signaling have been shown to lead to various placental-related complications including preeclampsia, miscarriage, and ectopic pregnancy [123,137,155]. Recently, Δ^9^-THC exposure was shown to disturb estradiol (E2) signaling in placental explants and BeWo cells. Concomitantly, Δ^9^-THC increased mRNA expression of aromatase (CYP19A1), the rate-limiting enzyme for E2 synthesis, and increased estrogen receptor alpha (ERα) expression (see Figure 2). The Δ^9^-THC-induced increase of aromatase was mediated by ERα-mediated signaling and dependent on CB1 activation, while Δ^9^-THC -induced expression of ERα was mediated via CB1 and CB2 receptors [156]. As such, cannabis consumption may impair placental steroidogenesis and endocrine signaling, key processes necessary for proper placentation and pregnancy. 

Recent toxicological studies have explored the role of the ECS in the placenta following exposure to exogenous cannabinoids. In fact, Δ^9^-THC significantly impacted placental ECS homeostasis by altering AEA levels and expression profile of its synthetic and catabolic enzymes, NAPE-PLD and FAAH [157]. While Δ^9^-THC exposure did not lead to any changes in placental 2-AG levels or the expression of DAGL, Δ^9^-THC differentially increased the expression of MAGL and decreased the other hydrolyzing enzymes, alpha-beta hydrolase domain-6 (ABHD6) and -12 (ABDH12) [158]. While these studies show that cannabinoids like Δ^9^-THC can affect placental ECS signaling, further investigations into how external cannabinoids impact placental development, function, and pregnancy outcomes as a result of cannabis consumption are warranted. 

## 4. Altered ECS Signaling during Fetal Development

The ECS also plays a crucial role in fetal development, from embryo implantation to neurodevelopment and peripheral organogenesis [40,107,159,160,161]. In mice, components of the ECS, such as CB2 and NAPE-PLD, FAAH, and CB1, have been detected from the one-, two- and four-cell stages of embryonic development, respectively [103,109]. AEA-CB1 signaling is involved in preimplantation embryo development, blastocyst activation and implantation [162,163]. Endocannabinoids have also been detected in fetal tissue, with levels of 2-AG being much higher than those of AEA [164]. While concentrations of AEA gradually increase throughout development until adult levels are reached [164], fetal levels of 2-AG are similar to those observed in young and in adult brains. Collectively, the evidence suggests that ECS component expression and activity must be tightly regulated from very early stages of development and throughout pregnancy to maintain offspring health [165,166].

In addition to short-term effects, external factors such as nutritional status, stress hormone levels, or exogenous compounds can adversely affect signaling systems including the ECS which may alter fetal programming and contribute to structural, functional, and behavioral abnormalities in the adult offspring [4,167,168,169,170,171,172]. Since the role of the ECS in neurodevelopment has been widely investigated, most studies that assess long-term effects of prenatal ECS disruption and cannabis exposure have focused on the nervous system. However, as mentioned before, the ECS is also involved in peripheral organogenesis and is present in multiple systems throughout development. While there is evidence that suggests that fetal ECS disruption may affect immune function [173,174], cardiac function [175] and liver development [61], for the purpose of this review only neurological, metabolic and reproductive impacts will be addressed.

### 4.1. Neurological Impacts

Components of the ECS are present in several brain structures from the very early stages of embryonic development [176,177,178]. The ECS has a precise and fundamental role in various aspects of neurodevelopment, including neuronal migration and axonal elongation, glia formation [176,179,180], neural stem cell proliferation and differentiation [181], orchestration of axonal migration and connectivity, and synaptogenesis [182,183,184].

**Figure 3:**
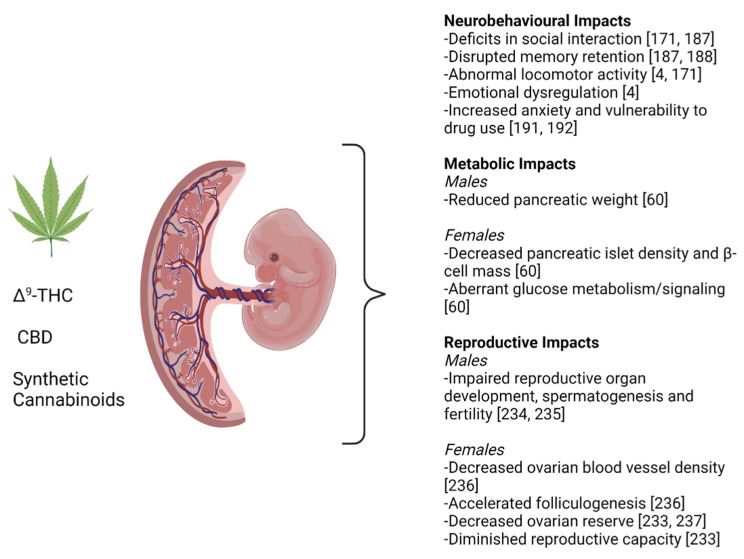
Animal models using cannabinoids, commercially available delta-9-tetrahydrocannabinol (Δ^9^-THC), cannabidiol (CBD) or synthetic cannabinoids have demonstrated a variety of neurobehavioral, metabolic and reproductive impacts. The various systemic effects of cannabis and its components, as determined using primarily rodent models, are summarized in this figure. Greater details about these studies can be found in the body of this review. This figure was created using BioRender.com accessed on 5 August 2021.

Both animal and human studies have demonstrated that prenatal cannabinoid exposure can result in long-lasting neurobehavioral abnormalities in the offspring [4,170,185,186]. In rodents, exposure to cannabinoids or cannabinoid receptor agonists (i.e., WIN55212, CP55940) during the perinatal period resulted in a wide range of effects such as deficits in social discrimination and interaction [171,187], disrupted memory retention [187,188,189], impaired object recognition [190], locomotor activity abnormalities, emotional dysregulation, and increased vulnerability to drugs of abuse [4,191,192] (see Figure 3). In particular, prenatal exposure to cannabinoids has been shown to alter the maturation of serotonergic [193], dopaminergic [194,195], GABAergic [196,197], glutamatergic [187,198,199], and opioidergic systems [192,200]. Little is known about the specific effects of CBD on the developing brain. In a study in which human induced pluripotent stem cells (hiPSC) were induced to differentiate into neuronal cells, thus mimicking developing fetal neurons, Δ^9^-THC (10 μM) promoted precocious neuronal and glial differentiation, while CBD was neurotoxic at the same concentration [201]. In a recent study conducted in mice, adult F1 offspring that were perinatally exposed to CBD exhibited differentially methylated loci in the cerebral cortex and hippocampus, as well as sex-specific increases in anxiety and memory [202]. Taken together, it is plausible that early life exposure to CBD may have lasting neurological impacts on adult offspring. In addition to direct cannabinoid exposure, inhibition of endocannabinoid metabolizing enzymes has also been shown to result in long-lasting effects [203,204]. For example, perinatal administration of the FAAH inhibitor URB597 led to depression-like symptoms and memory impairment in adult mice offspring [204]. 

In humans, major prospective longitudinal studies have found that cannabis-exposed offspring had reduced birthweight, slower growth, decreased head circumference [50], increased startle response, tremors, and deficient habituation to visual stimuli in neonates [205], as well as increased attention problems and signs of aggressive behavior in 18-month-old girls [206]. During childhood, cannabis-exposed offspring had diminished verbal and memory skills at 3 to 4 years of age [207,208], increased impulsivity and hyperactivity, as well as decreased concentration, IQ score, and verbal reasoning at 6 or 10 years of age [209,210]. As young adults (18 to 22 years of age), cannabis-exposed offspring presented with alterations in response inhibition and altered neural functioning during visuospatial working memory processing, as assessed by functional magnetic resonance imaging (fMRI) [186,211] (see Figure 1).

In addition to the similar outcomes observed between Δ^9^-THC and specific CB agonists, other evidence also suggests that the long-term effects of Δ^9^-THC on neurodevelopment are ECS-mediated. For example, it has been demonstrated that prenatal exposure to Δ^9^-THC leads to CB1 activation and neuronal rewiring through the degradation of the molecular effector superior cervical ganglion 10 (SCG10)/statmin 2, which is known to regulate microtubule dynamics in axons [212]. The erroneous synaptic rewiring of glutamatergic cortical neurons could partially explain drug-seeking behaviors observed in prenatally Δ^9^-THC-exposed adult offspring. Furthermore, prenatal Δ^9^-THC exposure of mice interfered with subcerebral projection neuron generation and altered corticospinal connectivity, producing long-lasting alterations in adult offspring motor function. Interestingly, CB1 null mice were resistant to these Δ^9^-THC-induced alterations [213].

Indeed, several comprehensive reviews have covered the neurodevelopmental and behavioral consequences of prenatal cannabis exposure and the involvement of the ECS [4,7,62,159,169,214,215,216,217]. While most studies have focused on the role of the ECS and its perturbation on the developing nervous system, other systems have received less attention. 

### 4.2. Metabolic Impacts

Despite its involvement in peripheral organogenesis, the long-term effects of fetal ECS disruption on organs other than the brain remain elusive. It has been shown that CB1 contributes to pancreatic islet formation and organization during fetal development, and that these effects are modulated by endogenous endocannabinoid levels in fetal tissue and circulation [125,160,164]. Additionally, CB2 has also been detected in the bovine fetal pancreas [218]. Given that Δ^9^-THC may have a direct effect on the developing pancreas through cannabinoid receptor interaction [160], and that impaired fetal growth has been associated with the development of type 2 diabetes [219], investigations into the metabolic effects associated with early life exposure to cannabis in the offspring are warranted. 

In a recent study conducted in rats, gestational Δ^9^-THC exposure significantly reduced birthweight and pancreatic weight in both males and females. However, at 5 months of age, only female offspring had decreased islet density and β-cell mass. In line with this effect, Δ^9^-THC-exposed female offspring also exhibited elevated blood glucose 5 min after a glucose challenge and an overall increased area under the curve for blood glucose. This was associated with significantly augmented serum insulin concentrations 15 min after the glucose challenge, suggesting that peripheral insulin resistance contributed to the observed glucose intolerance. Additionally, after an insulin challenge, Δ^9^-THC-exposed offspring demonstrated blunted pAkt [Ser473] activation in the soleus muscle, suggesting aberrant glucose metabolism signaling [60] (see Figure 3). Interestingly, CB1 activation has been shown to reduce pancreatic β-cell proliferation and impede insulin receptor activity in vitro [220,221], suggesting Δ^9^-THC-induced metabolic effects may be ECS-mediated.

Additionally, Δ^9^-THC has been shown to affect mitochondrial function in several tissues, including the placenta [148,149,222,223]. Human trophoblast cells exposed to Δ^9^-THC have diminished mitochondrial respiration and ATP-coupling due to decreased abundance of mitochondrial chain complex proteins [148], as well as increased mitochondrial fission and decreased mitochondrial membrane potential [149]. Given that fetal mitochondrial dysfunction has been linked to the onset of postnatal diseases such as type 2 diabetes and obesity [224], it is possible that Δ^9^-THC directly affects these organelles and disturbs metabolic homeostasis later in life.

Other stressors can impact fetal ECS signaling, which may in turn exert influences on metabolic homeostasis. Dias-Rocha and colleagues reported that maternal high-fat diet prior to mating, and during gestation and lactation, resulted in increased hypothalamic CB1 protein in male pups and increased hypothalamic CB2 protein in female pups at birth [225]. In brown adipose tissue, a maternal high-fat diet decreased CB1 in male pups and increased CB2 in female pups. Additionally, maternal high-fat diet adult offspring developed overweight phenotype, higher adiposity, and high-fat diet preference, independently of the sex, but only males presented hyperleptinemia and higher energy expenditure [225]. These studies suggest that fetal ECS disruption may have long-term effects on the offspring’s metabolic health, an aspect that has been largely overlooked.

### 4.3. Reproductive Impacts

While several studies have reported the direct effects of cannabis and Δ^9^-THC on both male and female fertility [226,227,228,229,230,231,232], very few have assessed the effect of prenatal disruption of the ECS on the reproductive health of exposed offspring.

Indeed, the expression of CB1 and CB2 has been detected in both male and female gonads, with detection as early as embryonic day 11.5 (E11.5) in mice [233]. Also in mice, perinatal exposure to high doses of cannabinoids (cannabidiol and cannabinol) has been shown to affect spermatogenesis and fertility in male offspring at 60–80 days of age [234]. Similarly, oral administration of high doses of CBD to pregnant rats throughout gestation and lactation resulted in decreased growth, delayed sexual maturation, neurobehavioral changes and adverse effects on male reproductive organ development and fertility in the offspring [235] (see Figure 3). Exposure to a CB2 agonist (JWH133), on the other hand, induced activation of the meiotic program in both male and female gonads in vitro. While gonocytes became arrested at early stages of prophase I, oocytes showed accelerated meiosis along with an increase in Ser-139-phosphorylated histone variant H2AX (γ-H2AX)-positive pachytene and diplotene cells and terminal deoxynucleotidyl transferase-mediated dUTP-fluorescein nick-end labeling (TUNEL)-positive cells, suggesting that DNA double-strand breaks were not correctly repaired, leading to oocyte apoptosis [233]. Administration of the same agonist to pregnant females resulted in a significant reduction of primordial and primary follicles in ovaries from newborn mice, as well as a diminished reproductive capacity as adults [233]. In a recent study, female rats that were exposed to Δ^9^-THC during gestation had accelerated folliculogenesis with apparent follicular development arrest at 6 months of age. Additionally, the ovaries of prenatally Δ^9^-THC-exposed offspring had reduced blood vessel density in association with decreased expression of the proangiogenic factor vascular endothelial growth factor (VEGF), and its receptor vascular endothelial growth factor receptor 2 (VEGFR2), as well as an increase in the antiangiogenic factor thrombospondin-1 (TSP1) [236]. In a similar study, prenatal exposure to the synthetic CB1/CB2 agonist, WIN55212, resulted in a decrease in ovarian reserve at 90 days of age (see Figure 3). The same decrease was not observed following co-administration with a CB1 inverse agonist (SR141716), suggesting that the effects of WIN55212 may be CB1-mediated. Interestingly, prenatal exposure to SR141716 alone resulted in an increase in the ovarian reserve compared to the vehicle group [237]. These studies suggest that the ECS plays an important part from the earliest stages of the reproductive process and that there is need for a deeper understanding of its complex roles in order to appreciate the functional consequences of prenatal perturbances.

## 5. Conclusions

Cannabis use during pregnancy has increased considerably in recent decades, particularly in developed countries. While several studies have linked prenatal cannabis use with negative birth outcomes, as well as long-lasting neurobehavioral alterations, the impact on other physiological aspects such as metabolic and reproductive health have received less attention. In addition, most studies have focused on Δ^9^-THC and synthetic cannabinoid receptor agonists, with very little research addressing the effects of CBD or endogenous ECS ligands. However, other cannabis components may also disrupt the fetal ECS and have long-term effects, highlighting the need for more whole cannabis exposure models, as well as experiments that consider other popular cannabis components such as CBD. Finally, more insight is needed regarding the mechanisms through which gestational exposure to cannabis constituents may result in persistent long-term alterations on the offspring

## References

[1] World Health Organization Cannabis. https://www.who.int/teams/mental-health-and-substance-use/alcohol-drugs-and-addictive-behaviours/drugs-psychoactive/cannabis.

[2] Statistics Canada National Cannabis Survey, Third Quarter 2019. https://www150.statcan.gc.ca/n1/daily-quotidien/191030/dq191030a-eng.htm.

[3] Alpar A., Di Marzo V., Harkany T. (2016). At the tip of an iceberg: Prenatal marijuana and Its possible relation to neuropsychiatric outcome in the offspring. Biol. Psychiatry.

[4] Campolongo P., Trezza V., Ratano P., Palmery M., Cuomo V. (2011). Developmental consequences of perinatal cannabis exposure: Behavioral and neuroendocrine effects in adult rodents. Psychopharmacology.

[5] Ebrahim S.H., Gfroerer J. (2003). Pregnancy-related substance use in the United States during 1996–1998. Obstet. Gynecol..

[6] McCabe J.E., Arndt S. (2012). Demographic and substance abuse trends among pregnant and non-pregnant women: Eleven years of treatment admission data. Matern. Child Health J..

[7] Wu C.S., Jew C.P., Lu H.C. (2011). Lasting impacts of prenatal cannabis exposure and the role of endogenous cannabinoids in the developing brain. Future Neurol..

[8] Ko J.Y., Farr S.L., Tong V.T., Creanga A.A., Callaghan W.M. (2015). Prevalence and patterns of marijuana use among pregnant and nonpregnant women of reproductive age. Am. J. Obstet. Gynecol..

[9] Roberson E.K., Patrick W.K., Hurwitz E.L. (2014). Marijuana use and maternal experiences of severe nausea during pregnancy in Hawai’i. Hawaii J. Med. Public Health.

[10] Westfall R.E., Janssen P.A., Lucas P., Capler R. (2006). Survey of medicinal cannabis use among childbearing women: Patterns of its use in pregnancy and retroactive self-assessment of its efficacy against ‘morning sickness’. Complement. Ther. Clin. Pract..

[11] Volkow N.D., Compton W.M., Wargo E.M. (2017). The risks of marijuana use during pregnancy. JAMA.

[12] Volkow N.D., Han B., Compton W.M., McCance-Katz E.F. (2019). Self-reported medical and nonmedical cannabis use among pregnant women in the United States. JAMA.

[13] Metz T.D., Borgelt L.M. (2018). Marijuana use in pregnancy and while breastfeeding. Obstet. Gynecol..

[14] Moore D.G., Turner J.D., Parrott A.C., Goodwin J.E., Fulton S.E., Min M.O., Fox H.C., Braddick F.M., Axelsson E.L., Lynch S. (2010). During pregnancy, recreational drug-using women stop taking ecstasy (3,4-methylenedioxy-N-methylamphetamine) and reduce alcohol consumption, but continue to smoke tobacco and cannabis: Initial findings from the Development and Infancy Study. J. Psychopharmacol..

[15] Corsi D.J., Hsu H., Weiss D., Fell D.B., Walker M. (2019). Trends and correlates of cannabis use in pregnancy: A population-based study in Ontario, Canada from 2012 to 2017. Can J. Public Health.

[16] Substance Abuse and Mental Health Services Administration 2019 National Survey on Drug Use and Health: Women. https://www.samhsa.gov/data/report/2019-nsduh-women.

[17] Leemaqz S.Y., Dekker G.A., McCowan L.M., Kenny L.C., Myers J.E., Simpson N.A., Poston L., Roberts C.T., Consortium S. (2016). Maternal marijuana use has independent effects on risk for spontaneous preterm birth but not other common late pregnancy complications. Reprod. Toxicol..

[18] Lozano J., Garcia-Algar O., Marchei E., Vall O., Monleon T., Giovannandrea R.D., Pichini S. (2007). Prevalence of gestational exposure to cannabis in a Mediterranean city by meconium analysis. Acta Paediatr..

[19] Metz T.D., Silver R.M., McMillin G.A., Allshouse A.A., Jensen T.L., Mansfield C., Heard K., Kinney G.L., Wymore E., Binswanger I.A. (2019). Prenatal marijuana use by self-report and umbilical cord sampling in a state with marijuana legalization. Obstet. Gynecol..

[20] PregnancyInfo. https://www.pregnancyinfo.ca/learn-more/.

[21] Vastis V., Vincent S., Metz T.D., Shea A.K. (2021). Are canadian cannabis dispensaries counselling pregnant women appropriately?. J. Obstet. Gynaecol. Can..

[22] Whiting P.F., Wolff R.F., Deshpande S., Di Nisio M., Duffy S., Hernandez A.V., Keurentjes J.C., Lang S., Misso K., Ryder S. (2015). Cannabinoids for Medical Use: A Systematic Review and Meta-analysis. JAMA.

[23] Sarrafpour S., Urits I., Powell J., Nguyen D., Callan J., Orhurhu V., Simopoulos T., Viswanath O., Kaye A.D., Kaye R.J. (2020). Considerations and implications of cannabidiol use during pregnancy. Curr. Pain Headache Rep..

[24] Appendino G., Chianese G., Taglialatela-Scafati O. (2011). Cannabinoids: Occurrence and medicinal chemistry. Curr. Med. Chem..

[25] Elsohly M.A., Slade D. (2005). Chemical constituents of marijuana: The complex mixture of natural cannabinoids. Life Sci..

[26] Dewey W.L. (1986). Cannabinoid pharmacology. Pharmacol. Rev..

[27] Gaoni Y., Mechoulam R. (1964). Isolation, structure, and partial synthesis of an active constituent of hashish. J. Am. Chem. Soc..

[28] Hollister L.E. (1986). Health aspects of cannabis. Pharmacol. Rev..

[29] Burgdorf J.R., Kilmer B., Pacula R.L. (2011). Heterogeneity in the composition of marijuana seized in California. Drug Alcohol Depend..

[30] ElSohly M.A., Mehmedic Z., Foster S., Gon C., Chandra S., Church J.C. (2016). Changes in cannabis potency over the last 2 decades (1995–2014): Analysis of current data in the United States. Biol. Psychiatry.

[31] ElSohly M.A., Ross S.A., Mehmedic Z., Arafat R., Yi B., Banahan B.F. (2000). Potency trends of delta9-THC and other cannabinoids in confiscated marijuana from 1980–1997. J. Forensic Sci..

[32] Mammen G., de Freitas L., Rehm J., Rueda S. (2017). Cannabinoid concentrations in Canada’s regulated medical cannabis industry. Addiction.

[33] Mehmedic Z., Chandra S., Slade D., Denham H., Foster S., Patel A.S., Ross S.A., Khan I.A., ElSohly M.A. (2010). Potency trends of Delta9-THC and other cannabinoids in confiscated cannabis preparations from 1993 to 2008. J. Forensic Sci..

[34] Health Canada About Cannabis. https://www.canada.ca/en/health-canada/services/drugs-medication/cannabis/about.html.

[35] Fonseca B.M., Costa M.A., Almada M., Correia-da-Silva G., Teixeira N.A. (2013). Endogenous cannabinoids revisited: A biochemistry perspective. Prostaglandins Other Lipid Mediat..

[36] Komorowski J., Stepien H. (2007). The role of the endocannabinoid system in the regulation of endocrine function and in the control of energy balance in humans. Postepy Hig. Med. Dosw. (Online).

[37] Habayeb O.M., Taylor A.H., Bell S.C., Taylor D.J., Konje J.C. (2008). Expression of the endocannabinoid system in human first trimester placenta and its role in trophoblast proliferation. Endocrinology.

[38] Lewis S.E., Rapino C., Di Tommaso M., Pucci M., Battista N., Paro R., Simon L., Lutton D., Maccarrone M. (2012). Differences in the endocannabinoid system of sperm from fertile and infertile men. PLoS ONE.

[39] Sun X., Dey S.K. (2012). Endocannabinoid signaling in female reproduction. ACS Chem. Neurosci..

[40] Taylor A.H., Ang C., Bell S.C., Konje J.C. (2007). The role of the endocannabinoid system in gametogenesis, implantation and early pregnancy. Hum. Reprod. Update.

[41] Singh S., Filion K.B., Abenhaim H.A., Eisenberg M.J. (2020). Prevalence and outcomes of prenatal recreational cannabis use in high-income countries: A scoping review. BJOG.

[42] Luke S., Hutcheon J., Kendall T. (2019). Cannabis use in pregnancy in British Columbia and selected birth outcomes. J. Obstet. Gynaecol. Can..

[43] Varner M.W., Silver R.M., Rowland Hogue C.J., Willinger M., Parker C.B., Thorsten V.R., Goldenberg R.L., Saade G.R., Dudley D.J., Coustan D. (2014). Association between stillbirth and illicit drug use and smoking during pregnancy. Obstet. Gynecol..

[44] Hatch E.E., Bracken M.B. (1986). Effect of marijuana use in pregnancy on fetal growth. Am. J. Epidemiol..

[45] Hayatbakhsh M.R., Flenady V.J., Gibbons K.S., Kingsbury A.M., Hurrion E., Mamun A.A., Najman J.M. (2012). Birth outcomes associated with cannabis use before and during pregnancy. Pediatr. Res..

[46] Metz T.D., Stickrath E.H. (2015). Marijuana use in pregnancy and lactation: A review of the evidence. Am. J. Obstet. Gynecol..

[47] Hurd Y.L., Wang X., Anderson V., Beck O., Minkoff H., Dow-Edwards D. (2005). Marijuana impairs growth in mid-gestation fetuses. Neurotoxicol. Teratol..

[48] Warshak C.R., Regan J., Moore B., Magner K., Kritzer S., Van Hook J. (2015). Association between marijuana use and adverse obstetrical and neonatal outcomes. J. Perinatol..

[49] Campbell E.E., Gilliland J., Dworatzek P.D.N., De Vrijer B., Penava D., Seabrook J.A. (2018). Socioeconomic status and adverse birth outcomes: A population-based Canadian sample. J. Biosoc. Sci..

[50] El Marroun H., Tiemeier H., Steegers E.A., Jaddoe V.W., Hofman A., Verhulst F.C., van den Brink W., Huizink A.C. (2009). Intrauterine cannabis exposure affects fetal growth trajectories: The Generation R Study. J. Am. Acad. Child Adolesc. Psychiatry.

[51] Gabrhelik R., Mahic M., Lund I.O., Bramness J., Selmer R., Skovlund E., Handal M., Skurtveit S. (2021). Cannabis use during pregnancy and risk of adverse birth outcomes: A longitudinal cohort study. Eur. Addict. Res..

[52] Gunn J.K., Rosales C.B., Center K.E., Nunez A., Gibson S.J., Christ C., Ehiri J.E. (2016). Prenatal exposure to cannabis and maternal and child health outcomes: A systematic review and meta-analysis. BMJ Open.

[53] Conner S.N., Carter E.B., Tuuli M.G., Macones G.A., Cahill A.G. (2015). Maternal marijuana use and neonatal morbidity. Am. J. Obstet. Gynecol..

[54] Mark K., Desai A., Terplan M. (2016). Marijuana use and pregnancy: Prevalence, associated characteristics, and birth outcomes. Arch. Womens Ment. Health.

[55] van Gelder M.M., Reefhuis J., Caton A.R., Werler M.M., Druschel C.M., Roeleveld N., National Birth Defects Prevention Study (2010). Characteristics of pregnant illicit drug users and associations between cannabis use and perinatal outcome in a population-based study. Drug Alcohol Depend..

[56] Government of Canada Canadian Cannabis Survey 2020: Summary. https://www.canada.ca/en/health-canada/services/drugs-medication/cannabis/research-data/canadian-cannabis-survey-2020-summary.html.

[57] Abel E.L., Tan S.E., Subramanian M. (1987). Effects of delta 9-tetrahydrocannabinol, phenobarbital, and their combination on pregnancy and offspring in rats. Teratology.

[58] Wenger T., Fragkakis G., Giannikou P., Yiannikakis N. (1997). The effects of prenatally administered endogenous cannabinoid on rat offspring. Pharmacol. Biochem. Behav..

[59] Benevenuto S.G., Domenico M.D., Martins M.A., Costa N.S., de Souza A.R., Costa J.L., Tavares M.F., Dolhnikoff M., Veras M.M. (2017). Recreational use of marijuana during pregnancy and negative gestational and fetal outcomes: An experimental study in mice. Toxicology.

[60] Gillies R., Lee K., Vanin S., Laviolette S.R., Holloway A.C., Arany E., Hardy D.B. (2020). Maternal exposure to Delta9-tetrahydrocannabinol impairs female offspring glucose homeostasis and endocrine pancreatic development in the rat. Reprod. Toxicol..

[61] Natale B.V., Gustin K.N., Lee K., Holloway A.C., Laviolette S.R., Natale D.R.C., Hardy D.B. (2020). Delta9-tetrahydrocannabinol exposure during rat pregnancy leads to symmetrical fetal growth restriction and labyrinth-specific vascular defects in the placenta. Sci. Rep..

[62] Richardson K.A., Hester A.K., McLemore G.L. (2016). Prenatal cannabis exposure—The “first hit” to the endocannabinoid system. Neurotoxicol. Teratol..

[63] Bellocchio L., Cervino C., Pasquali R., Pagotto U. (2008). The endocannabinoid system and energy metabolism. J. Neuroendocrinol..

[64] Burstein S.H., Zurier R.B. (2009). Cannabinoids, endocannabinoids, and related analogs in inflammation. AAPS J..

[65] Gomes T.M., Dias da Silva D., Carmo H., Carvalho F., Silva J.P. (2020). Epigenetics and the endocannabinoid system signaling: An intricate interplay modulating neurodevelopment. Pharmacol. Res..

[66] Walker O.S., Holloway A.C., Raha S. (2019). The role of the endocannabinoid system in female reproductive tissues. J. Ovarian Res..

[67] Woodhams S.G., Sagar D.R., Burston J.J., Chapman V. (2015). The role of the endocannabinoid system in pain. Handb. Exp. Pharmacol..

[68] Lu H.C., Mackie K. (2016). An introduction to the endogenous cannabinoid system. Biol. Psychiatry.

[69] Battista N., Di Tommaso M., Bari M., Maccarrone M. (2012). The endocannabinoid system: An overview. Front. Behav. Neurosci..

[70] Devane W.A., Hanus L., Breuer A., Pertwee R.G., Stevenson L.A., Griffin G., Gibson D., Mandelbaum A., Etinger A., Mechoulam R. (1992). Isolation and structure of a brain constituent that binds to the cannabinoid receptor. Science.

[71] Mechoulam R., Ben-Shabat S., Hanus L., Ligumsky M., Kaminski N.E., Schatz A.R., Gopher A., Almog S., Martin B.R., Compton D.R. (1995). Identification of an endogenous 2-monoglyceride, present in canine gut, that binds to cannabinoid receptors. Biochem. Pharmacol..

[72] Rodriguez de Fonseca F., Del Arco I., Bermudez-Silva F.J., Bilbao A., Cippitelli A., Navarro M. (2005). The endocannabinoid system: Physiology and pharmacology. Alcohol Alcohol..

[73] Pertwee R.G., Howlett A.C., Abood M.E., Alexander S.P., Di Marzo V., Elphick M.R., Greasley P.J., Hansen H.S., Kunos G., Mackie K. (2010). International Union of Basic and Clinical Pharmacology. LXXIX. Cannabinoid receptors and their ligands: Beyond CB(1) and CB(2). Pharmacol. Rev..

[74] Maccarrone M., Dainese E., Oddi S. (2010). Intracellular trafficking of anandamide: New concepts for signaling. Trends Biochem. Sci..

[75] Oddi S., Fezza F., Pasquariello N., De Simone C., Rapino C., Dainese E., Finazzi-Agro A., Maccarrone M. (2008). Evidence for the intracellular accumulation of anandamide in adiposomes. Cell. Mol. Life Sci..

[76] Lucas C.J., Galettis P., Schneider J. (2018). The pharmacokinetics and the pharmacodynamics of cannabinoids. Br. J. Clin. Pharmacol..

[77] Breijyeh Z., Jubeh B., Bufo S.A., Karaman R., Scrano L. (2021). Cannabis: A toxin-producing plant with potential therapeutic uses. Toxins.

[78] Kennedy E.K.C., Perono G.A., Nemez D.B., Holloway A.C., Thomas P.J., Letcher R., Marvin C., Stetefeld J., Stout J., Peters O. (2020). Increasing cannabis use and importance as an environmental contaminant mixture and associated risks to exposed biota: A review. Crit. Rev. Environ. Sci. Technol..

[79] Laprairie R.B., Bagher A.M., Kelly M.E., Denovan-Wright E.M. (2015). Cannabidiol is a negative allosteric modulator of the cannabinoid CB1 receptor. Br. J. Pharmacol..

[80] Ujvary I., Hanus L. (2016). Human metabolites of cannabidiol: A review on their formation, biological activity, and relevance in therapy. Cannabis Cannabinoid Res..

[81] Hourani W., Alexander S.P.H. (2018). Cannabinoid ligands, receptors and enzymes: Pharmacological tools and therapeutic potential. Brain Neurosci. Adv..

[82] Pertwee R.G. (1997). Pharmacology of cannabinoid CB1 and CB2 receptors. Pharmacol. Ther..

[83] Chakravarti B., Ravi J., Ganju R.K. (2014). Cannabinoids as therapeutic agents in cancer: Current status and future implications. Oncotarget.

[84] Herrera B., Carracedo A., Diez-Zaera M., Gomez del Pulgar T., Guzman M., Velasco G. (2006). The CB2 cannabinoid receptor signals apoptosis via ceramide-dependent activation of the mitochondrial intrinsic pathway. Exp. Cell Res..

[85] Pacher P., Batkai S., Kunos G. (2006). The endocannabinoid system as an emerging target of pharmacotherapy. Pharmacol. Rev..

[86] Zou S., Kumar U. (2018). Cannabinoid receptors and the endocannabinoid system: Signaling and function in the central nervous system. Int. J. Mol. Sci.

[87] Ryberg E., Larsson N., Sjogren S., Hjorth S., Hermansson N.O., Leonova J., Elebring T., Nilsson K., Drmota T., Greasley P.J. (2007). The orphan receptor GPR55 is a novel cannabinoid receptor. Br. J. Pharmacol..

[88] Sawzdargo M., Nguyen T., Lee D.K., Lynch K.R., Cheng R., Heng H.H., George S.R., O’Dowd B.F. (1999). Identification and cloning of three novel human G protein-coupled receptor genes GPR52, PsiGPR53 and GPR55: GPR55 is extensively expressed in human brain. Mol. Brain Res..

[89] Lauckner J.E., Jensen J.B., Chen H.Y., Lu H.C., Hille B., Mackie K. (2008). GPR55 is a cannabinoid receptor that increases intracellular calcium and inhibits M current. Proc. Natl. Acad. Sci. USA.

[90] Marichal-Cancino B.A., Fajardo-Valdez A., Ruiz-Contreras A.E., Mendez-Diaz M., Prospero-Garcia O. (2017). Advances in the physiology of GPR55 in the central nervous system. Curr. Neuropharmacol..

[91] Romero-Zerbo S.Y., Rafacho A., Diaz-Arteaga A., Suarez J., Quesada I., Imbernon M., Ross R.A., Dieguez C., Rodriguez de Fonseca F., Nogueiras R. (2011). A role for the putative cannabinoid receptor GPR55 in the islets of Langerhans. J. Endocrinol..

[92] Simcocks A.C., O′Keefe L., Jenkin K.A., Mathai M.L., Hryciw D.H., McAinch A.J. (2014). A potential role for GPR55 in the regulation of energy homeostasis. Drug Discov. Today.

[93] Lowin T., Straub R.H. (2015). Cannabinoid-based drugs targeting CB1 and TRPV1, the sympathetic nervous system, and arthritis. Arthritis Res. Ther..

[94] Petrosino S., Schiano Moriello A., Cerrato S., Fusco M., Puigdemont A., De Petrocellis L., Di Marzo V. (2016). The anti-inflammatory mediator palmitoylethanolamide enhances the levels of 2-arachidonoyl-glycerol and potentiates its actions at TRPV1 cation channels. Br. J. Pharmacol..

[95] Ross R.A. (2003). Anandamide and vanilloid TRPV1 receptors. Br. J. Pharmacol..

[96] Ahluwalia J., Urban L., Bevan S., Nagy I. (2003). Anandamide regulates neuropeptide release from capsaicin-sensitive primary sensory neurons by activating both the cannabinoid 1 receptor and the vanilloid receptor 1 In Vitro. Eur. J. Neurosci..

[97] Anand U., Otto W.R., Sanchez-Herrera D., Facer P., Yiangou Y., Korchev Y., Birch R., Benham C., Bountra C., Chessell I.P. (2008). Cannabinoid receptor CB2 localisation and agonist-mediated inhibition of capsaicin responses in human sensory neurons. Pain.

[98] Pistis M., Melis M. (2010). From surface to nuclear receptors: The endocannabinoid family extends its assets. Curr. Med. Chem..

[99] Rouzer C.A., Marnett L.J. (2011). Endocannabinoid oxygenation by cyclooxygenases, lipoxygenases, and cytochromes P450: Cross-talk between the eicosanoid and endocannabinoid signaling pathways. Chem. Rev..

[100] Zelasko S., Arnold W.R., Das A. (2015). Endocannabinoid metabolism by cytochrome P450 monooxygenases. Prostaglandins Other Lipid Mediat..

[101] El-Talatini M.R., Taylor A.H., Elson J.C., Brown L., Davidson A.C., Konje J.C. (2009). Localisation and function of the endocannabinoid system in the human ovary. PLoS ONE.

[102] Schuel H., Burkman L.J., Lippes J., Crickard K., Forester E., Piomelli D., Giuffrida A. (2002). N-Acylethanolamines in human reproductive fluids. Chem. Phys. Lipids.

[103] Paria B.C., Das S.K., Dey S.K. (1995). The preimplantation mouse embryo is a target for cannabinoid ligand-receptor signaling. Proc. Natl. Acad. Sci. USA.

[104] Das S.K., Paria B.C., Chakraborty I., Dey S.K. (1995). Cannabinoid ligand-receptor signaling in the mouse uterus. Proc. Natl. Acad. Sci. USA.

[105] Scotchie J.G., Savaris R.F., Martin C.E., Young S.L. (2015). Endocannabinoid regulation in human endometrium across the menstrual cycle. Reprod. Sci..

[106] Park B., Gibbons H.M., Mitchell M.D., Glassa M. (2003). Identification of the CB1 cannabinoid receptor and fatty acid amide hydrolase (FAAH) in the human placenta. Placenta.

[107] Correa F., Wolfson M.L., Valchi P., Aisemberg J., Franchi A.M. (2016). Endocannabinoid system and pregnancy. Reproduction.

[108] Schmid P.C., Paria B.C., Krebsbach R.J., Schmid H.H., Dey S.K. (1997). Changes in anandamide levels in mouse uterus are associated with uterine receptivity for embryo implantation. Proc. Natl. Acad. Sci. USA.

[109] Wang H., Xie H., Guo Y., Zhang H., Takahashi T., Kingsley P.J., Marnett L.J., Das S.K., Cravatt B.F., Dey S.K. (2006). Fatty acid amide hydrolase deficiency limits early pregnancy events. J. Clin. Investig..

[110] Xie H., Sun X., Piao Y., Jegga A.G., Handwerger S., Ko M.S., Dey S.K. (2012). Silencing or amplification of endocannabinoid signaling in blastocysts via CB1 compromises trophoblast cell migration. J. Biol. Chem..

[111] Yang Z.M., Paria B.C., Dey S.K. (1996). Activation of brain-type cannabinoid receptors interferes with preimplantation mouse embryo development. Biol. Reprod..

[112] Burton G.J., Fowden A.L. (2015). The placenta: A multifaceted, transient organ. Philos. Trans. R. Soc. Lond. B Biol. Sci..

[113] Knofler M., Haider S., Saleh L., Pollheimer J., Gamage T., James J. (2019). Human placenta and trophoblast development: Key molecular mechanisms and model systems. Cell. Mol. Life Sci..

[114] Costa M.A. (2016). The endocannabinoid system: A novel player in human placentation. Reprod. Toxicol..

[115] Ezechukwu H.C., Diya C.A., Shrestha N., Hryciw D.H. (2020). Role for endocannabinoids in early pregnancy: Recent advances and the effects of cannabis use. Am. J. Physiol. Endocrinol. Metab..

[116] Maia J., Fonseca B.M., Teixeira N., Correia-da-Silva G. (2020). The fundamental role of the endocannabinoid system in endometrium and placenta: Implications in pathophysiological aspects of uterine and pregnancy disorders. Hum. Reprod. Update.

[117] Helliwell R.J., Chamley L.W., Blake-Palmer K., Mitchell M.D., Wu J., Kearn C.S., Glass M. (2004). Characterization of the endocannabinoid system in early human pregnancy. J. Clin. Endocrinol. Metab..

[118] Kenney S.P., Kekuda R., Prasad P.D., Leibach F.H., Devoe L.D., Ganapathy V. (1999). Cannabinoid receptors and their role in the regulation of the serotonin transporter in human placenta. Am. J. Obstet. Gynecol..

[119] Aban C., Leguizamon G.F., Cella M., Damiano A., Franchi A.M., Farina M.G. (2013). Differential expression of endocannabinoid system in normal and preeclamptic placentas: Effects on nitric oxide synthesis. Placenta.

[120] Costa M.A., Fonseca B.M., Keating E., Teixeira N.A., Correia-da-Silva G. (2014). 2-arachidonoylglycerol effects in cytotrophoblasts: Metabolic enzymes expression and apoptosis in BeWo cells. Reproduction.

[121] Costa M.A., Keating E., Fonseca B.M., Teixeira N.A., Correia-da-Silva G. (2015). 2-Arachidonoylglycerol impairs human cytotrophoblast cells syncytialization: Influence of endocannabinoid signalling in placental development. Mol. Cell Endocrinol..

[122] Sun X., Xie H., Yang J., Wang H., Bradshaw H.B., Dey S.K. (2010). Endocannabinoid signaling directs differentiation of trophoblast cell lineages and placentation. Proc. Natl. Acad. Sci. USA.

[123] Trabucco E., Acone G., Marenna A., Pierantoni R., Cacciola G., Chioccarelli T., Mackie K., Fasano S., Colacurci N., Meccariello R. (2009). Endocannabinoid system in first trimester placenta: Low FAAH and high CB1 expression characterize spontaneous miscarriage. Placenta.

[124] Marczylo T.H., Lam P.M., Amoako A.A., Konje J.C. (2010). Anandamide levels in human female reproductive tissues: Solid-phase extraction and measurement by ultraperformance liquid chromatography tandem mass spectrometry. Anal. Biochem..

[125] Brocato B., Zoerner A.A., Janjetovic Z., Skobowiat C., Gupta S., Moore B.M., Slominski A., Zhang J., Schenone M., Phinehas R. (2013). Endocannabinoid crosstalk between placenta and maternal fat in a baboon model (*Papio* spp.) of obesity. Placenta.

[126] Fonseca B.M., Correia-da-Silva G., Taylor A.H., Lam P.M., Marczylo T.H., Konje J.C., Teixeira N.A. (2012). Characterisation of the endocannabinoid system in rat haemochorial placenta. Reprod. Toxicol..

[127] Costa M.A., Fonseca B.M., Keating E., Teixeira N.A., Correia-da-Silva G. (2014). Transient receptor potential vanilloid 1 is expressed in human cytotrophoblasts: Induction of cell apoptosis and impairment of syncytialization. Int. J. Biochem. Cell Biol..

[128] Kremshofer J., Siwetz M., Berghold V.M., Lang I., Huppertz B., Gauster M. (2015). A role for GPR55 in human placental venous endothelial cells. Histochem. Cell Biol..

[129] Aban C.E., Accialini P.L., Etcheverry T., Leguizamon G.F., Martinez N.A., Farina M.G. (2018). Crosstalk between nitric oxide and endocannabinoid signaling pathways in normal and pathological placentation. Front. Physiol..

[130] Costa M.A., Fonseca B.M., Teixeira N.A., Correia-da-Silva G. (2015). The endocannabinoid anandamide induces apoptosis in cytotrophoblast cells: Involvement of both mitochondrial and death receptor pathways. Placenta.

[131] Almada M., Costa L., Fonseca B., Alves P., Braga J., Goncalves D., Teixeira N., Correia-da-Silva G. (2020). The endocannabinoid 2-arachidonoylglycerol promotes endoplasmic reticulum stress in placental cells. Reproduction.

[132] Etcheverry T., Accialini P., Palligas M., Loureiro F., Saraco N., Martinez N., Farina M. (2021). Endocannabinoid signaling impairs syncytialization: Using flow cytometry to evaluate forskolin-induced cell fusion. Placenta.

[133] Szilagyi J.T., Composto-Wahler G.M., Joseph L.B., Wang B., Rosen T., Laskin J.D., Aleksunes L.M. (2019). Anandamide down-regulates placental transporter expression through CB2 receptor-mediated inhibition of cAMP synthesis. Pharmacol. Res..

[134] Habayeb O.M., Taylor A.H., Evans M.D., Cooke M.S., Taylor D.J., Bell S.C., Konje J.C. (2004). Plasma levels of the endocannabinoid anandamide in women—A potential role in pregnancy maintenance and labor?. J. Clin. Endocrinol. Metab..

[135] Maccarrone M., Valensise H., Bari M., Lazzarin N., Romanini C., Finazzi-Agro A. (2000). Relation between decreased anandamide hydrolase concentrations in human lymphocytes and miscarriage. Lancet.

[136] Maccarrone M., Bisogno T., Valensise H., Lazzarin N., Fezza F., Manna C., Di Marzo V., Finazzi-Agro A. (2002). Low fatty acid amide hydrolase and high anandamide levels are associated with failure to achieve an ongoing pregnancy after IVF and embryo transfer. Mol. Hum. Reprod..

[137] Molvarec A., Fugedi G., Szabo E., Stenczer B., Walentin S., Rigo J. (2015). Decreased circulating anandamide levels in preeclampsia. Hypertens. Res..

[138] Fugedi G., Molnar M., Rigo J., Schonleber J., Kovalszky I., Molvarec A. (2014). Increased placental expression of cannabinoid receptor 1 in preeclampsia: An observational study. BMC Pregnancy Childbirth.

[139] Thompson R., DeJong K., Lo J. (2019). Marijuana use in pregnancy: A review. Obstet. Gynecol. Surv..

[140] Bailey J.R., Cunny H.C., Paule M.G., Slikker W. (1987). Fetal disposition of delta 9-tetrahydrocannabinol (THC) during late pregnancy in the rhesus monkey. Toxicol. Appl. Pharmacol..

[141] Blackard C., Tennes K. (1984). Human placental transfer of cannabinoids. N. Engl. J. Med..

[142] Hutchings D.E., Martin B.R., Gamagaris Z., Miller N., Fico T. (1989). Plasma concentrations of delta-9-tetrahydrocannabinol in dams and fetuses following acute or multiple prenatal dosing in rats. Life Sci..

[143] Khare M., Taylor A.H., Konje J.C., Bell S.C. (2006). Delta9-tetrahydrocannabinol inhibits cytotrophoblast cell proliferation and modulates gene transcription. Mol. Hum. Reprod..

[144] Walker O.S., Ragos R., Gurm H., Lapierre M., May L.L., Raha S. (2020). Delta-9-tetrahydrocannabinol disrupts mitochondrial function and attenuates syncytialization in human placental BeWo cells. Physiol. Rep..

[145] Costa M.A., Fonseca B.M., Marques F., Teixeira N.A., Correia-da-Silva G. (2015). The psychoactive compound of Cannabis sativa, Delta(9)-tetrahydrocannabinol (THC) inhibits the human trophoblast cell turnover. Toxicology.

[146] Almada M., Alves P., Fonseca B.M., Carvalho F., Queiros C.R., Gaspar H., Amaral C., Teixeira N.A., Correia-da-Silva G. (2020). Synthetic cannabinoids JWH-018, JWH-122, UR-144 and the phytocannabinoid THC activate apoptosis in placental cells. Toxicol. Lett..

[147] Almada M., Costa L., Fonseca B.M., Amaral C., Teixeira N., Correia-da-Silva G. (2017). The synthetic cannabinoid WIN-55,212 induced-apoptosis in cytotrophoblasts cells by a mechanism dependent on CB1 receptor. Toxicology.

[148] Lojpur T., Easton Z., Raez-Villanueva S., Laviolette S., Holloway A.C., Hardy D.B. (2019). Delta9-Tetrahydrocannabinol leads to endoplasmic reticulum stress and mitochondrial dysfunction in human BeWo trophoblasts. Reprod. Toxicol..

[149] Walker O.S., Gurm H., Sharma R., Verma N., May L.L., Raha S. (2021). Delta-9-tetrahydrocannabinol inhibits invasion of HTR8/SVneo human extravillous trophoblast cells and negatively impacts mitochondrial function. Sci. Rep..

[150] Araujo J.R., Goncalves P., Martel F. (2009). Effect of cannabinoids upon the uptake of folic acid by BeWo cells. Pharmacology.

[151] Keating E., Goncalves P., Campos I., Costa F., Martel F. (2009). Folic acid uptake by the human syncytiotrophoblast: Interference by pharmacotherapy, drugs of abuse and pathological conditions. Reprod. Toxicol..

[152] Almada M., Amaral C., Oliveira A., Fernandes P.A., Ramos M.J., Fonseca B.M., Correia-da-Silva G., Teixeira N. (2020). Cannabidiol (CBD) but not tetrahydrocannabinol (THC) dysregulate in vitro decidualization of human endometrial stromal cells by disruption of estrogen signaling. Reprod. Toxicol..

[153] Feinshtein V., Erez O., Ben-Zvi Z., Eshkoli T., Sheizaf B., Sheiner E., Holcberg G. (2013). Cannabidiol enhances xenobiotic permeability through the human placental barrier by direct inhibition of breast cancer resistance protein: An ex vivo study. Am. J. Obstet. Gynecol..

[154] Costa M.A. (2016). The endocrine function of human placenta: An overview. Reprod. Biomed. Online.

[155] Berkane N., Liere P., Oudinet J.P., Hertig A., Lefevre G., Pluchino N., Schumacher M., Chabbert-Buffet N. (2017). From pregnancy to preeclampsia: A key role for estrogens. Endocr. Rev..

[156] Maia J., Almada M., Midao L., Fonseca B.M., Braga J., Goncalves D., Teixeira N., Correia-da-Silva G. (2020). The cannabinoid delta-9-tetrahydrocannabinol disrupts estrogen signaling in human placenta. Toxicol. Sci..

[157] Maia J., Midao L., Cunha S.C., Almada M., Fonseca B.M., Braga J., Goncalves D., Teixeira N., Correia-da-Silva G. (2019). Effects of cannabis tetrahydrocannabinol on endocannabinoid homeostasis in human placenta. Arch. Toxicol..

[158] Maia J., Fonseca B.M., Cunha S.C., Braga J., Goncalves D., Teixeira N., Correia-da-Silva G. (2020). Impact of tetrahydrocannabinol on the endocannabinoid 2-arachidonoylglycerol metabolism: ABHD6 and ABHD12 as novel players in human placenta. Biochim. Biophys. Acta Mol. Cell Biol. Lipids.

[159] Maccarrone M., Guzman M., Mackie K., Doherty P., Harkany T. (2014). Programming of neural cells by (endo)cannabinoids: From physiological rules to emerging therapies. Nat. Rev. Neurosci..

[160] Malenczyk K., Keimpema E., Piscitelli F., Calvigioni D., Bjorklund P., Mackie K., Di Marzo V., Hokfelt T.G., Dobrzyn A., Harkany T. (2015). Fetal endocannabinoids orchestrate the organization of pancreatic islet microarchitecture. Proc. Natl. Acad. Sci. USA.

[161] Oltrabella F., Melgoza A., Nguyen B., Guo S. (2017). Role of the endocannabinoid system in vertebrates: Emphasis on the zebrafish model. Dev. Growth Differ..

[162] Paria B.C., Deutsch D.D., Dey S.K. (1996). The uterus is a potential site for anandamide synthesis and hydrolysis: Differential profiles of anandamide synthase and hydrolase activities in the mouse uterus during the periimplantation period. Mol. Reprod. Dev..

[163] Paria B.C., Song H., Wang X., Schmid P.C., Krebsbach R.J., Schmid H.H., Bonner T.I., Zimmer A., Dey S.K. (2001). Dysregulated cannabinoid signaling disrupts uterine receptivity for embryo implantation. J. Biol. Chem..

[164] Berrendero F., Sepe N., Ramos J.A., Di Marzo V., Fernandez-Ruiz J.J. (1999). Analysis of cannabinoid receptor binding and mRNA expression and endogenous cannabinoid contents in the developing rat brain during late gestation and early postnatal period. Synapse.

[165] Maccarrone M., Finazzi-Agro A. (2004). Anandamide hydrolase: A guardian angel of human reproduction?. Trends Pharmacol. Sci..

[166] Wang J., Paria B.C., Dey S.K., Armant D.R. (1999). Stage-specific excitation of cannabinoid receptor exhibits differential effects on mouse embryonic development. Biol. Reprod..

[167] Barker D.J. (1998). In utero programming of chronic disease. Clin. Sci. (Lond.).

[168] Bernard C., Milh M., Morozov Y.M., Ben-Ari Y., Freund T.F., Gozlan H. (2005). Altering cannabinoid signaling during development disrupts neuronal activity. Proc. Natl. Acad. Sci. USA.

[169] Calvigioni D., Hurd Y.L., Harkany T., Keimpema E. (2014). Neuronal substrates and functional consequences of prenatal cannabis exposure. Eur. Child Adolesc. Psychiatry.

[170] Fried P.A., Smith A.M. (2001). A literature review of the consequences of prenatal marihuana exposure. An emerging theme of a deficiency in aspects of executive function. Neurotoxicol. Teratol..

[171] Navarro M., Rubio P., de Fonseca F.R. (1995). Behavioural consequences of maternal exposure to natural cannabinoids in rats. Psychopharmacology.

[172] Schneider M. (2009). Cannabis use in pregnancy and early life and its consequences: Animal models. Eur. Arch. Psychiatry Clin. Neurosci..

[173] Dong C., Chen J., Harrington A., Vinod K.Y., Hegde M.L., Hegde V.L. (2019). Cannabinoid exposure during pregnancy and its impact on immune function. Cell. Mol. Life Sci..

[174] Zumbrun E.E., Sido J.M., Nagarkatti P.S., Nagarkatti M. (2015). Epigenetic regulation of immunological alterations following prenatal exposure to marijuana cannabinoids and its long term consequences in offspring. J. Neuroimmune Pharmacol..

[175] Lee K., Laviolette S.R., Hardy D.B. (2021). Exposure to Delta9-tetrahydrocannabinol during rat pregnancy leads to impaired cardiac dysfunction in postnatal life. Pediatr. Res..

[176] Fride E., Gobshtis N., Dahan H., Weller A., Giuffrida A., Ben-Shabat S. (2009). Chapter 6 The endocannabinoid system during development: Emphasis on perinatal events and delayed effects. Vitam. Horm..

[177] Harkany T., Keimpema E., Barabas K., Mulder J. (2008). Endocannabinoid functions controlling neuronal specification during brain development. Mol. Cell Endocrinol..

[178] Jutras-Aswad D., DiNieri J.A., Harkany T., Hurd Y.L. (2009). Neurobiological consequences of maternal cannabis on human fetal development and its neuropsychiatric outcome. Eur. Arch. Psychiatry Clin. Neurosci..

[179] Fernandez-Ruiz J., Gomez M., Hernandez M., de Miguel R., Ramos J.A. (2004). Cannabinoids and gene expression during brain development. Neurotox. Res..

[180] Mulder J., Aguado T., Keimpema E., Barabas K., Ballester Rosado C.J., Nguyen L., Monory K., Marsicano G., Di Marzo V., Hurd Y.L. (2008). Endocannabinoid signaling controls pyramidal cell specification and long-range axon patterning. Proc. Natl. Acad. Sci. USA.

[181] Galve-Roperh I., Chiurchiu V., Diaz-Alonso J., Bari M., Guzman M., Maccarrone M. (2013). Cannabinoid receptor signaling in progenitor/stem cell proliferation and differentiation. Prog. Lipid Res..

[182] Berghuis P., Rajnicek A.M., Morozov Y.M., Ross R.A., Mulder J., Urban G.M., Monory K., Marsicano G., Matteoli M., Canty A. (2007). Hardwiring the brain: Endocannabinoids shape neuronal connectivity. Science.

[183] Diaz-Alonso J., Guzman M., Galve-Roperh I. (2012). Endocannabinoids via CB(1) receptors act as neurogenic niche cues during cortical development. Philos. Trans. R. Soc. Lond. B Biol. Sci..

[184] Gaffuri A.L., Ladarre D., Lenkei Z. (2012). Type-1 cannabinoid receptor signaling in neuronal development. Pharmacology.

[185] Goldschmidt L., Richardson G.A., Cornelius M.D., Day N.L. (2004). Prenatal marijuana and alcohol exposure and academic achievement at age 10. Neurotoxicol. Teratol..

[186] Smith A.M., Fried P.A., Hogan M.J., Cameron I. (2006). Effects of prenatal marijuana on visuospatial working memory: An fMRI study in young adults. Neurotoxicol. Teratol..

[187] Campolongo P., Trezza V., Cassano T., Gaetani S., Morgese M.G., Ubaldi M., Soverchia L., Antonelli T., Ferraro L., Massi M. (2007). Perinatal exposure to delta-9-tetrahydrocannabinol causes enduring cognitive deficits associated with alteration of cortical gene expression and neurotransmission in rats. Addict. Biol..

[188] Mereu G., Fa M., Ferraro L., Cagiano R., Antonelli T., Tattoli M., Ghiglieri V., Tanganelli S., Gessa G.L., Cuomo V. (2003). Prenatal exposure to a cannabinoid agonist produces memory deficits linked to dysfunction in hippocampal long-term potentiation and glutamate release. Proc. Natl. Acad. Sci. USA.

[189] Silva L., Zhao N., Popp S., Dow-Edwards D. (2012). Prenatal tetrahydrocannabinol (THC) alters cognitive function and amphetamine response from weaning to adulthood in the rat. Neurotoxicol. Teratol..

[190] O’Shea M., McGregor I.S., Mallet P.E. (2006). Repeated cannabinoid exposure during perinatal, adolescent or early adult ages produces similar longlasting deficits in object recognition and reduced social interaction in rats. J. Psychopharmacol..

[191] Spano M.S., Ellgren M., Wang X., Hurd Y.L. (2007). Prenatal cannabis exposure increases heroin seeking with allostatic changes in limbic enkephalin systems in adulthood. Biol. Psychiatry.

[192] Vela G., Martin S., Garcia-Gil L., Crespo J.A., Ruiz-Gayo M., Fernandez-Ruiz J.J., Garcia-Lecumberri C., Pelaprat D., Fuentes J.A., Ramos J.A. (1998). Maternal exposure to delta9-tetrahydrocannabinol facilitates morphine self-administration behavior and changes regional binding to central mu opioid receptors in adult offspring female rats. Brain Res..

[193] Molina-Holgado F., Alvarez F.J., Gonzalez I., Antonio M.T., Leret M.L. (1997). Maternal exposure to delta 9-tetrahydrocannabinol (delta 9-THC) alters indolamine levels and turnover in adult male and female rat brain regions. Brain Res. Bull..

[194] DiNieri J.A., Wang X., Szutorisz H., Spano S.M., Kaur J., Casaccia P., Dow-Edwards D., Hurd Y.L. (2011). Maternal cannabis use alters ventral striatal dopamine D2 gene regulation in the offspring. Biol. Psychiatry.

[195] Wang X., Dow-Edwards D., Anderson V., Minkoff H., Hurd Y.L. (2004). In utero marijuana exposure associated with abnormal amygdala dopamine D2 gene expression in the human fetus. Biol. Psychiatry.

[196] Garcia-Gil L., de Miguel R., Romero J., Perez A., Ramos J.A., Fernandez-Ruiz J.J. (1999). Perinatal delta9-tetrahydrocannabinol exposure augmented the magnitude of motor inhibition caused by GABA(B), but not GABA(A), receptor agonists in adult rats. Neurotoxicol. Teratol..

[197] Saez T.M., Aronne M.P., Caltana L., Brusco A.H. (2014). Prenatal exposure to the CB1 and CB2 cannabinoid receptor agonist WIN 55,212-2 alters migration of early-born glutamatergic neurons and GABAergic interneurons in the rat cerebral cortex. J. Neurochem..

[198] Antonelli T., Tanganelli S., Tomasini M.C., Finetti S., Trabace L., Steardo L., Sabino V., Carratu M.R., Cuomo V., Ferraro L. (2004). Long-term effects on cortical glutamate release induced by prenatal exposure to the cannabinoid receptor agonist (R)-(+)-[2,3-dihydro-5-methyl-3-(4-morpholinyl-methyl)pyrrolo[1,2,3-de]-1,4-benzo xazin-6-yl]-1-naphthalenylmethanone: An in vivo microdialysis study in the awake rat. Neuroscience.

[199] Suarez I., Bodega G., Rubio M., Fernandez-Ruiz J.J., Ramos J.A., Fernandez B. (2004). Prenatal cannabinoid exposure down- regulates glutamate transporter expressions (GLAST and EAAC1) in the rat cerebellum. Dev. Neurosci..

[200] Kumar A.M., Haney M., Becker T., Thompson M.L., Kream R.M., Miczek K. (1990). Effect of early exposure to delta-9-tetrahydrocannabinol on the levels of opioid peptides, gonadotropin-releasing hormone and substance P in the adult male rat brain. Brain Res..

[201] Miranda C.C., Barata T., Vaz S.H., Ferreira C., Quintas A., Bekman E.P. (2020). hiPSC-based model of prenatal exposure to cannabinoids: Effect on neuronal differentiation. Front. Mol. Neurosci..

[202] Wanner N.M., Colwell M., Drown C., Faulk C. (2021). Developmental cannabidiol exposure increases anxiety and modifies genome-wide brain DNA methylation in adult female mice. Clin. Epigenetics.

[203] Schlosburg J.E., Blankman J.L., Long J.Z., Nomura D.K., Pan B., Kinsey S.G., Nguyen P.T., Ramesh D., Booker L., Burston J.J. (2010). Chronic monoacylglycerol lipase blockade causes functional antagonism of the endocannabinoid system. Nat. Neurosci..

[204] Wu C.S., Morgan D., Jew C.P., Haskins C., Andrews M.J., Leishman E., Spencer C.M., Czyzyk T., Bradshaw H., Mackie K. (2014). Long-term consequences of perinatal fatty acid amino hydrolase inhibition. Br. J. Pharmacol..

[205] Fried P.A., Makin J.E. (1987). Neonatal behavioural correlates of prenatal exposure to marihuana, cigarettes and alcohol in a low risk population. Neurotoxicol. Teratol..

[206] El Marroun H., Hudziak J.J., Tiemeier H., Creemers H., Steegers E.A., Jaddoe V.W., Hofman A., Verhulst F.C., van den Brink W., Huizink A.C. (2011). Intrauterine cannabis exposure leads to more aggressive behavior and attention problems in 18-month-old girls. Drug Alcohol Depend..

[207] Fried P.A., Watkinson B. (1990). 36- and 48-month neurobehavioral follow-up of children prenatally exposed to marijuana, cigarettes, and alcohol. J. Dev. Behav. Pediatr..

[208] Day N.L., Richardson G.A., Goldschmidt L., Robles N., Taylor P.M., Stoffer D.S., Cornelius M.D., Geva D. (1994). Effect of prenatal marijuana exposure on the cognitive development of offspring at age three. Neurotoxicol. Teratol..

[209] Goldschmidt L., Richardson G.A., Willford J., Day N.L. (2008). Prenatal marijuana exposure and intelligence test performance at age 6. J. Am. Acad. Child Adolesc. Psychiatry.

[210] Richardson G.A., Ryan C., Willford J., Day N.L., Goldschmidt L. (2002). Prenatal alcohol and marijuana exposure: Effects on neuropsychological outcomes at 10 years. Neurotoxicol. Teratol..

[211] Smith A.M., Fried P.A., Hogan M.J., Cameron I. (2004). Effects of prenatal marijuana on response inhibition: An fMRI study of young adults. Neurotoxicol. Teratol..

[212] Tortoriello G., Morris C.V., Alpar A., Fuzik J., Shirran S.L., Calvigioni D., Keimpema E., Botting C.H., Reinecke K., Herdegen T. (2014). Miswiring the brain: Delta9-tetrahydrocannabinol disrupts cortical development by inducing an SCG10/stathmin-2 degradation pathway. EMBO J..

[213] de Salas-Quiroga A., Diaz-Alonso J., Garcia-Rincon D., Remmers F., Vega D., Gomez-Canas M., Lutz B., Guzman M., Galve-Roperh I. (2015). Prenatal exposure to cannabinoids evokes long-lasting functional alterations by targeting CB1 receptors on developing cortical neurons. Proc. Natl. Acad. Sci. USA.

[214] Fride E. (2008). Multiple roles for the endocannabinoid system during the earliest stages of life: Pre- and postnatal development. J. Neuroendocrinol..

[215] Harkany T., Guzman M., Galve-Roperh I., Berghuis P., Devi L.A., Mackie K. (2007). The emerging functions of endocannabinoid signaling during CNS development. Trends Pharmacol. Sci..

[216] Higuera-Matas A., Ucha M., Ambrosio E. (2015). Long-term consequences of perinatal and adolescent cannabinoid exposure on neural and psychological processes. Neurosci. Biobehav. Rev..

[217] Scheyer A.F., Melis M., Trezza V., Manzoni O.J.J. (2019). Consequences of perinatal cannabis exposure. Trends Neurosci..

[218] Dall’Aglio C., Polisca A., Cappai M.G., Mercati F., Troisi A., Pirino C., Scocco P., Maranesi M. (2017). Immunohistochemistry detected and localized cannabinoid receptor type 2 in bovine fetal pancreas at late gestation. Eur. J. Histochem..

[219] Hales C.N., Barker D.J. (1992). Type 2 (non-insulin-dependent) diabetes mellitus: The thrifty phenotype hypothesis. Diabetologia.

[220] Bazwinsky-Wutschke I., Zipprich A., Dehghani F. (2019). Endocannabinoid system in hepatic glucose metabolism, fatty liver disease, and cirrhosis. Int. J. Mol. Sci..

[221] Kim W., Doyle M.E., Liu Z., Lao Q., Shin Y.K., Carlson O.D., Kim H.S., Thomas S., Napora J.K., Lee E.K. (2011). Cannabinoids inhibit insulin receptor signaling in pancreatic beta-cells. Diabetes.

[222] Athanasiou A., Clarke A.B., Turner A.E., Kumaran N.M., Vakilpour S., Smith P.A., Bagiokou D., Bradshaw T.D., Westwell A.D., Fang L. (2007). Cannabinoid receptor agonists are mitochondrial inhibitors: A unified hypothesis of how cannabinoids modulate mitochondrial function and induce cell death. Biochem. Biophys. Res. Commun..

[223] Mendizabal-Zubiaga J., Melser S., Benard G., Ramos A., Reguero L., Arrabal S., Elezgarai I., Gerrikagoitia I., Suarez J., Rodriguez De Fonseca F. (2016). Cannabinoid CB1 receptors are localized in striated muscle mitochondria and regulate mitochondrial respiration. Front. Physiol..

[224] Bansal A., Rashid C., Simmons R.A., Morio B., Pénicaud L., Rigoulet M. (2019). Chapter 14—Impact of fetal programming on mitochondrial function and susceptibility to obesity and type 2 diabetes. Mitochondria in Obesity and Type 2 Diabetes.

[225] Dias-Rocha C.P., Almeida M.M., Santana E.M., Costa J.C.B., Franco J.G., Pazos-Moura C.C., Trevenzoli I.H. (2018). Maternal high-fat diet induces sex-specific endocannabinoid system changes in newborn rats and programs adiposity, energy expenditure and food preference in adulthood. J. Nutr. Biochem..

[226] Brents L.K. (2016). Marijuana, the endocannabinoid system and the female reproductive system. Yale J. Biol. Med..

[227] Gundersen T.D., Jorgensen N., Andersson A.M., Bang A.K., Nordkap L., Skakkebaek N.E., Priskorn L., Juul A., Jensen T.K. (2015). Association between use of marijuana and male reproductive hormones and semen quality: A study among 1,215 healthy young men. Am. J. Epidemiol..

[228] Mendelson J.H., Mello N.K., Ellingboe J., Skupny A.S., Lex B.W., Griffin M. (1986). Marihuana smoking suppresses luteinizing hormone in women. J. Pharmacol. Exp. Ther..

[229] Reich R., Laufer N., Lewysohn O., Cordova T., Ayalon D., Tsafriri A. (1982). In vitro effects of cannabinoids on follicular function in the rat. Biol. Reprod..

[230] Rosenkrantz H., Grant R.J., Fleischman R.W., Baker J.R. (1986). Marihuana-induced embryotoxicity in the rabbit. Fundam. Appl. Toxicol..

[231] Treinen K.A., Sneeden J.L., Heindel J.J. (1993). Specific inhibition of FSH-stimulated cAMP accumulation by delta 9-tetrahydrocannabinol in cultured rat granulosa cells. Toxicol. Appl. Pharmacol..

[232] Smith C.G., Almirez R.G., Berenberg J., Asch R.H. (1983). Tolerance develops to the disruptive effects of delta 9-tetrahydrocannabinol on primate menstrual cycle. Science.

[233] De Domenico E., Todaro F., Rossi G., Dolci S., Geremia R., Rossi P., Grimaldi P. (2017). Overactive type 2 cannabinoid receptor induces meiosis in fetal gonads and impairs ovarian reserve. Cell Death Dis..

[234] Dalterio S.L., deRooij D.G. (1986). Maternal cannabinoid exposure. Effects on spermatogenesis in male offspring. Int. J. Androl..

[235] GW Pharmaceuticals Plc Reports Fiscal Fourth Quarter and Year-End 2018 Financial Results and Operational Progress. https://ir.gwpharm.com/news-releases/news-release-details/gw-pharmaceuticals-plc-reports-fiscal-fourth-quarter-and-year-0.

[236] Martinez-Pena A.A., Lee K., Petrik J.J., Hardy D.B., Holloway A.C. (2021). Gestational exposure to Delta(9)-THC impacts ovarian follicular dynamics and angiogenesis in adulthood in Wistar rats. J. Dev. Orig. Health Dis..

[237] Castel P., Barbier M., Poumerol E., Mandon-Pepin B., Tassistro V., Lepidi H., Pelissier-Alicot A.L., Manzoni O.J., Courbiere B. (2020). Prenatal cannabinoid exposure alters the ovarian reserve in adult offspring of rats. Arch. Toxicol..

